# League of Remembrance

**Published:** 1924-06

**Authors:** 


### THE LEAGUE OF REMEMBRANCE.

A reception was held by the League of Remembrance at their headquarters, 1 Marlborough Gate, W., during the afternoon of May 13. Princess Beatrice, the President of the League, was present and afterwards presided at a meeting of the Medical Advisory Board. Among those present were Countess Bathurst, Viscount and Viscountess Lee of Fareham, Blanche Lady Dodsworth, Lady Edmonstone, Lady Cowan, Mrs. Graham Glasgow, Miss Louise Dale, Mrs. E. H. Gibson, C.B.E. (the honorary general manager), Mr. John Murray, C.V.O. (the chairman), Sir William Arbuthnot Lane, Lady Lane, &c:

### What the League Does.

It may be of interest here to give a short account of the objects and work of the League. Its main objects are " to perpetuate the memory of the War Hospital Supply Depot movement of the years 1914-1919. To maintain the supply of medical and surgical requisites and clothing to . . . institutions and organisations engaged in the promotion of health or social welfare, the materials being provided by the organisations requiring assistance, the League making them up into whatever dressings or garments they may require without any charge for its services. To provide financial help to widows, children and other dependents of officers who have fallen or been incapacitated in the war. To provide and maintain a home for the League at which members can meet and keep in touch with the progress and changes in hospital work and other matters connected with the health and social life of the nation "—indeed a splendid and practical form of " Remembrance."

### Overseas Activities.

The work of the League for the past year, reviewed by Mrs. E. H. Gibson, showed that during the year 174 hospitals and infant welfare centres had been helped by the League, representing the making of 45,000 garments and dressings. The League has entirely equipped the new Maternity Ward at the Westminster Hospital and has been able to execute weekly orders for some of the principal London Hospitals. The amount of work is increasing so rapidly that the number of paid heads of workrooms, all officers' widows, has been increased from six to thirteen and larger premises are already becoming necessary. Entertainments have been given to ex-Service men still in hospital and have been greatly appreciated by them. It is intended by its organisers and members that the League shall be the nucleus of an organisation available for expansion in cases of national emergency. The League has a widespread overseas branch with representatives in Brazil, New Zealand, Uganda, U.S.A., France, Ceylon and Australia. It sent out in January a snowball letter which has been on a journey round the world and has been returned with the following verse adorning its envelope :—

" Oh, Postman, treat it gently for it's travelled many miles,
It's parched and torn but still afloat, and bound for British Isles ;
It's bound where would that I were bound, and bears from distant lands,
The best of fortune to the League and all for which it stands."

